# Converting habits of antibiotic use for respiratory tract infections in German primary care (CHANGE-3) - process evaluation of a complex intervention

**DOI:** 10.1186/s12875-020-01351-2

**Published:** 2020-12-19

**Authors:** R. Poß-Doering, L. Kuehn, M. Kamradt, K. Glassen, Th. Fleischhauer, P. Kaufmann-Kolle, M. Koeppen, A. Wollny, A. Altiner, M. Wensing

**Affiliations:** 1 Department of General Practice and Health Services Research, University Hospital Heidelberg, Im Neuenheimer Feld 130.3, 69120 Heidelberg, Germany; 2 aQua Institut, Maschmuehlenweg 8-10, 37073 Goettingen, Germany; 3 University Medical Center Rostock, Institute of General Practice, Doberaner Str. 142, 18057 Rostock, Germany

**Keywords:** Process evaluation, Mixed methods, Respiratory tract infections, Antibiotics, Prevention, Primary care, Germany

## Abstract

**Background:**

Antimicrobial resistance remains a global challenge. In Germany, the national health agenda supports measures that enhance the appropriate, guideline-oriented use of antibiotics. The study “Converting Habits of Antibiotic Use for Respiratory Tract Infections in German Primary Care (CHANGE-3)” aimed at a sustainable reduction of antimicrobial resistance through converting patterns of prescribing practice and use of antibiotics and an increase in health literacy in primary care patients, practice teams, and in the general public. Embedded in a cluster-randomized trial of a multifaceted implementation program, a process evaluation focused on the uptake of program components to assess the fidelity of the implementation program in the CHANGE-3 study and to understand utilization of its educational components.

**Methods:**

A mix of qualitative and quantitative methods was used. Semi-structured telephone interviews were conducted with General Practitioners, Medical Assistants, patients treated for respiratory tract infection and outreach visitors who had carried out individual outreach visits. A two-wave written survey (T1: 5 months after start, T2: 16 months after start) was conducted in general practitioners and medical assistants. Qualitative data were analyzed using thematic framework analysis. Descriptive statistics were used to analyze survey data.

**Results:**

Uptake of intervention components was heterogenous. Across all components, the uptake reported by General Practitioners varied from 20 to 88% at T1 and 31 to 63% at T2. Medical Assistants reported uptake from 22 to 70% at T1 and 6 to 69% at T2. Paper-based components could by and large be integrated in daily practice (64 to 90% in T1; 41 to 93% in T2), but uptake of digital components was low. A one-time outreach visit provided thematic information and feedback regarding actual prescribing, but due to time constraints were received with reluctance by practice teams. Patients were largely unaware of program components, but assumed that information and education could promote health literacy regarding antibiotics use.

**Conclusions:**

The process evaluation contributed to understanding the applicability of the delivered educational components with regards to the appropriate use of antibiotics. Future research efforts need to identify the best mode of delivery to reach the targeted population.

**Trial registration:**

ISRCTN, ISRCTN15061174. Registered 13 July 2018 – Retrospectively registered

**Supplementary Information:**

The online version contains supplementary material available at 10.1186/s12875-020-01351-2.

## Background

Antimicrobial resistance remains a topic on the global health agenda as even after decades of scientific research and improvement programs on the appropriate and guideline-oriented use of antibiotics, there still is ample room for improvement [1]. In Germany, the national agenda has reinforced policies to restrain antibiotics prescribing [2]. Here, about 90% of the used antibiotics are prescribed in ambulatory care, mainly by General Practitioners (GPs) [3], most commonly during 41% of the consultations for acute respiratory tract infections (ARTI) and with 52% of those in accordance with guideline recommendations [4]. Such prescribing patterns can be based on a deceptive safety culture and a misinterpretation of patient expectations [5, 6].

The appropriate use of antibiotics has been the topic of health services research in recent years, which has contributed to a growing evidence base regarding effects of educational interventions for physicians. These contributions include findings related to peer exchange mechanisms and audit and feedback processes [7], provider-patient communication and shared decision making [8, 9], internet-based training [10] and addressing the complete practice team to optimize organizational processes [11]. Research indicated that strategies using educational components such as communication skills training, point-of-care testing or patient information pamphlets can support lower antibiotics prescription rates [12–15]. A recent systematic review summarized the evidence on the effectiveness of interventions aiming to reduce antibiotic prescriptions in primary care and concluded that generalizability of observed effects of implementation strategies was limited due to heterogeneous designs and outcome measures [12].

The project “Converting Habits of Antibiotic Use for Respiratory Tract Infections in German Primary Care (CHANGE-3)” aimed to sustainably promote the conversion of patterns of antibiotics prescribing and use and increase health literacy in primary care practice teams, patients and the general public. In a novel approach, a regional public campaign was combined with a practice team intervention providing educational interventions for general practitioners and medical assistants. The process evaluation (PE) conducted alongside the project used a mix of methods to assess uptake of the program and understand the utilization of its components. By applying the Theoretical Domains Framework (TDF) [16, 17], which provides domains that may support understanding of potential determinants of behaviour, the PE aimed to provide insight into the contribution of the educational interventions to outcomes and to inform future implementation strategies.

## Objectives

The objective of this study was to examine the contribution of the educational interventions in the CHANGE-3 project to changes in antibiotics use patterns. More specifically, it aimed to assess

1. whether the program reached the targeted individuals and was conducted as planned
2. perceived effects on daily practice of healthcare delivery to patients with ARTI, on decision making of patient and provider, and the perceived increase in health literacy in the targeted population
3. the impact of program components and context factors on health services for patients with ARTI to explore patient expectations, and organizational aspects.

## Methods

### Study design

**Fig. 1:**
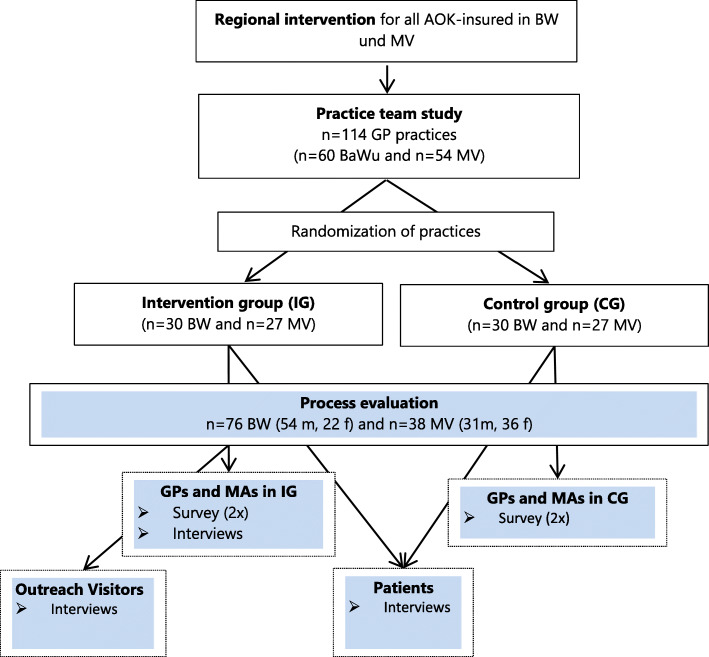
CHANGE-3 Study Design: Regional intervention with embedded cluster-RCT and process evaluation (GP = General Practitioner, MA = Medical Assistant, BW = Baden-Wuerttemberg, MV = Mecklenburg-Vorpommern; IG = intervention group; CG = control group, m = male, f = female)

The CHANGE-3 study was conducted as a two-armed cluster-RCT with a practice team intervention and a control group and a regional intervention which addressed the general public through a multi-media awareness campaign. Outcomes of the implementation program were assessed in a nested cluster-randomized trial, in which all practices in the intervention group and patients were exposed to educational interventions as well as to the public awareness campaign. Randomization and allocation of the 114 participating practices in the practice team study (Baden Wuerttemberg (BW) *n* = 60, Mecklenburg Western Pomerania (MV) *n* = 54) to the two arms of the study was done by statisticians and concealed from all others in the project. Additional randomization of participants in the process evaluation was not applicable. Figure 1 illustrates the overall study design for CHANGE-3 with the highlighted process evaluation design.

A PE was conducted alongside the implementation program as a prospective observational study. In a complex intervention, a PE aims at understanding the functioning of the intervention by investigating the uptake of intervention components, mechanisms of impact and contextual factors [18]. The PE in CHANGE-3 used a mix of qualitative and quantitative methods in a convergent-parallel design and focused on the effectiveness of the educational outreach visits plus a public campaign - as compared to public campaign only.

### Implementation program

The implementation program has been described elsewhere [19] and is briefly summarized here. General practices in the intervention group received an educational quality improvement program between September 2018 and March 2019 in order to strengthen health literacy competencies in GPs, MAs and patients. On single practice level, the intervention components addressed the healthcare provider team (GPs and MAs). A central component of the practice-team intervention was an outreach visit by experienced outreach visitors (OVs) who presented general information on antibiotic prescribing and the CHANGE-3 project. Outreach visits are considered to be a credible instrument in continuous medical education [20] and might be chosen for changes which are considered to be difficult to achieve [21]. They can improve the care delivered to patients and provide small to moderate changes in prescribing patterns [22]. A feedback report detailing the practice-individual antibiotic prescribing for ARTI was discussed. The reports were generated from claims data provided by the statutory health insurance AOK, reflected prescribing from the fourth quarter in 2016, and allowed for comparisons to other practice entities. All practices in the intervention group had access to an e-learning module focusing on strategies for provider-patient communication. They also received a tablet pc which was intended to deliver thematically focused information about the use of antibiotics to patients in waiting areas via a pre-installed e-learning application. Tablet-based apps have been used in diverse contexts to provide educational information [23–25]. All material referring to the regional intervention addressing the general public were to be introduced to the practice team during the outreach visit as well.

In addition, a regional intervention ran from September 2018 to January 2020. It consisted of a web- and paper-based public awareness campaign addressing children, adolescents, parents, young and middle-aged adults as well as the older population with a multi-media approach using digital and paper-based information material. Printed background information detailing consequences of inappropriate antibiotics use and possible alternatives for treatment of ARTI were provided to the intervention and to the control group. The core component of the campaign was a study-specific website, which was developed by communication design students and specialists in collaboration with primary care experts to provide evidence-based information regarding the use of antibiotics and alternative treatments for ARTI to the public and to health professionals [26]. Through an embedded web shop, supplies of the material used in the awareness campaign (printed educational flyers for patients, educational posters for practices, a coloring book and plush toy for small children, a comic for school-age children, and the educational magazine COLD for waiting areas) could be ordered free of charge by practices in the intervention and control group and by non-participating practices.

In the first phase of the study, all practices in the intervention group received a starter set comprising the paper-based information material and in the second phase they also received a small number of the COLD magazine and the plush toy.

**Fig. 2:**
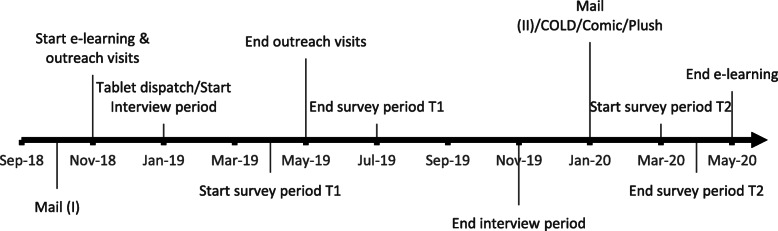
CHANGE-3 timeline. Chronological illustration of study component implementation and data measurement periods of the applied process evaluation

Table 1 lists all components offered to participants in the CHANGE-3 study. Figure 2 displays the chronology of study components and data collection periods of the process evaluation.

**Table 1:** Categories of intervention components offered in the CHANGE-3 study and their modes of delivery

Category	Intervention group	Control group	Available via website shop
Non-digital components			
for participating GPs, MAs and their patients	Flyer^a^ (German and other languages)	N/A	Flyer^a^(German and other languages)
	Poster	N/A	Poster
	COLD magazine^b^	N/A	COLD magazine^b^
	Plush toy^b^	N/A	Plush toy^b^
	Comic^b^	N/A	Comic^b^
	Coloring book	N/A	Coloring book
for healthcare provider teams	Outreach visit^c^	N/A	N/A
	Feedback report (two times)	N/A	N/A
	Printed thematic background information via postal mail	Printed thematic background information via postal mail	N/A
Digital components			
for GPs, MAs, patients and general public	Educational website providing information related to the use of antibiotics and alternatives	Educational website providing information related to the use of antibiotics and alternatives	N/A
for patients	Tablet devices providing educational app	N/A	N/A
for healthcare provider teams	e-learning platform providing communication training	N/A	N/A

^a^Available in German, English, French, Turkish, Vietnamese, Russian, Arab

^b^Component only offered in second half of the intervention period

^c^ Component only offered in first half of the intervention period (one time)

### Study population

The intervention in the CHANGE-3 study (educational practice team study) was intended for 114 participating general care practices in BW (n = 60) and MV (n = 54) [19]. The PE was based on a two-wave survey and qualitative interview research. The survey was aimed at GPs and MAs in the intervention and the control group, qualitative interview research was aimed at GPs and MAs in the intervention group, patients in both groups and at OVs.

To be eligible for participation in the PE, ambulatory practices needed to be located in MV or BW, allocated to the intervention group or the control group of the practice team study of CHANGE-3, and belong to the medical specialist group of GPs. MAs eligible to participate in the process evaluation were employees of participating practices. Patients eligible to participate in the PE were patients of the participating general practices who had been treated in these practices for respiratory tract infection during the intervention period. Further inclusion criteria were written and spoken German language skills and > 18 years old. Patients living in care facilities, suffering from dementia, incapacitated mentally or contacting a practice during locum care were excluded. Patients meeting the specified inclusion criteria were approached in the GP practice by the local healthcare providers. OVs eligible to participate in the PE had carried out at least five outreach visits to participating practices. All participants in the process evaluation had to be aged 18 years and above with full command of written and spoken German. Written informed consent was considered a pre-requisite for participation and were obtained separately for survey and interview participation during the recruitment process.

Due to the drop-out of 5 practices, data could be collected in 109 participating practices. All physicians (*n* = 132) and a proportion of MAs in the intervention group and in the control group contacted for general study participation received information about the PE and were invited to participate in it. MA participation was limited to two per practice with the intention to limit imbalance in the sample and to stay within the available reimbursement budget.

### Recruitment and sampling

The PE followed a purposive sampling strategy with regard to region (BW and MV) and participants’ sex to recruit a balanced sample of telephone interview participants. Recruitment was supported and initiated by the aQua Institut, Goettingen, and carried out by the study team at the Department of General Practice and Health Services Research, University Hospital Heidelberg. Using an opt-in approach, participating practices in the intervention and in the control group were asked to support patient recruitment along a structured process. The practice team handed out written information about the study and the PE, as well as a contact form to be sent to the Department of General Practice and Health Services Research, University Hospital Heidelberg via fax by the respective practice team. OVs who had conducted a minimum of five outreach visits each were contacted through the study team supported by the aQua Institut, Goettingen.

All potential interview participants received written information about the PE, as well as a contact form to be sent by fax or e-mail to the study team. After interest in participation in the PE was stated via the initial contact form, the informed consent form was mailed out. Members of the study team contacted interested GPs, MAs, patients and outreach visitors by phone to provide detailed information verbally. Upon receiving the informed consent form signed by the recruited participant, interview dates were scheduled.

### Measures

The survey questionnaires and the interview guides were based on the Theoretical Domains Framework (TDF) which is a comprehensive, theoretical framework that provides a basis to identify determinants of behaviour patterns of key actors to identify barriers of implementation [16, 17]. Due to their strong representation in the interview data, findings related to the three TDF domains (I) Environmental context and resources, (II) Social/professional role and identity and (III) Beliefs about consequences are reported here to focus on aspects regarding the regional intervention with multi-media content and e-learning, the practice team intervention with outreach visit and feedback report and on personal perceptions regarding the provision of health services to patients with ARTI. In addition, the survey focused on the general uptake of the intervention components. Survey and interview study included questions referring to socio-demographic aspects like age, gender, years of working experience and characteristics of the working environment.

Tailored questions for the interviews and survey questionnaires covered (a) the uptake and perceived impact of intervention components by participants with a focus on care for patients with ARTI, (b) consequences of the intervention for daily practice as well as on decision support (Social/professional role and identity), (c) the perceived impact of diverse context factors on health services for patients with acute respiratory tract infections (Environmental context and resources), and additionally, the written questionnaires contained items about (d) perceptions of patients’ expectations regarding antibiotics prescribing (Beliefs about consequences).

### Survey study

Data was generated via a two-time survey (GPs, MAs) across the intervention and control group. Survey periods were April to July 2019 (T1) and March to April 2020 (T2). The T1 survey questionnaire had a total of 5 sections. Section A focused on aspects regarding the regional intervention with multimedia content (web-based public campaign). Section B related to the practice team intervention with outreach visit, e-learning and feedback report (intervention group only). Section C comprised items referring to contextual factors (structural practice factors; motivational factors for prescribing decisions). Section D focused on personal attitudes and perceptions regarding the provision of health services to ARTI patients. In Section E, socio-demographic and practice characteristics were asked for (gender, age (year of birth), professional experience, size and location of practice (number of patients per quarter; urban, rural), participation in continued training, type of practice (single or group-practice, health centre, other). The T2 survey questionnaire comprised 3 sections. All items in section A referred to the intervention components. Section B focused on attitudes and perspectives on patient care and section C asked for a subset of the socio-demographic and practice characteristics (year of birth, gender, professional experience and number of patients seen per quarter). The questionnaires for MAs were adapted in section D to also ask about patient expectations. T1 and T2 questionnaires for the control group followed the same structure, but contained no items referring to the practice team intervention.

Written, paper-based questionnaires were mailed to participating GPs and MAs across study groups at two separate points in time (T1, T2). To increase the response rate, a reminder was sent out for T1 three weeks after the mailing date. Survey items were scored on a 5-point Likert scale ranging from 1 (strongly disagree) to 5 (strongly agree). Questionnaires were returned in an enclosed postage-paid envelope directly to a study nurse at the Department of General Practice and Health Services Research, University Hospital Heidelberg who registered and pseudonymized them. Data generated via the paper-based questionnaires were transformed into electronic data sets.

### Interview study

In the intervention group, semi-structured guide-based telephone interviews were conducted with GPs, MAs, patients and OVs. In the control group, interviews were exclusively applied to patients. In depth interviews were executed in two phases. In the first phase, GPs, MAs and patients were interviewed by two experienced researchers (RPD, MK) regarding the overall objectives of the PE. In the second phase, OVs, GPs who had received an outreach visit (VGPs) and one expert in the field were interviewed regarding their perceptions about outreach visits by a junior researcher (TF).

All interview guides were developed by the interprofessional team of researchers (Health Services Research, Public Health, General Practice) (see Additional files 1, 2, 3 and 4 for translated versions) and were based on constructs of the TDF, a literature review and pre-defined research questions. All interviews were audio-recorded, pseudonymized and transcribed verbatim.

### Data analysis

All data collected from the survey, the interview study, and socio-demographic questionnaires were pseudonymized prior to analysis, electronically saved and stored on secure servers at the Department of General Practice and Health Services Research, University Hospital, Heidelberg.

In the survey study, descriptive statistics were used to (a) characterize the study sample, (b) assess fidelity and perceived impacts on daily practice of healthcare delivery, and (c) impact of context factors on health services for patients by tabulating measures of the empirical distribution. According to the level of variables, means, standard deviations (SDs), and absolute or relative frequencies are reported. The general uptake of devices is reported by transforming ordinal five-point Likert scale levels into binary variables. The statistic software IBM SPSS 25 (IBM, Armonk, NY, USA) was used to conduct quantitative analysis.

**Fig. 3:**
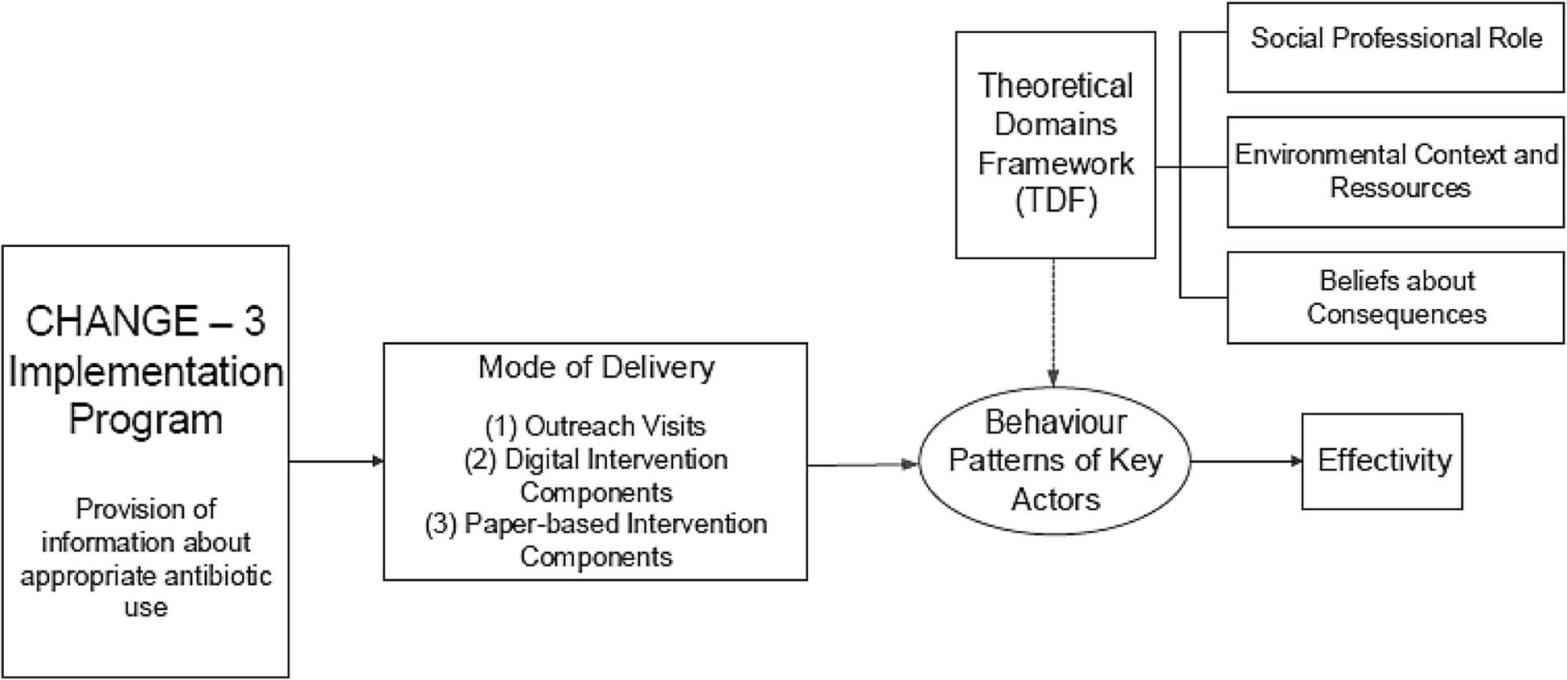
Methodical approach based on domains of Theoretical Domains Framework

In the interview study, all interviews were audio-recorded and transcribed verbatim. Thematic framework analysis was used to classify and organize data deductively according to a priori defined categories derived from the TDF [16] to identify determinants of practice which influence health care delivery to ARTI patients in general practices, with regard to the educational components of the intervention and with regard to converting patterns of antibiotic use. Categories were also identified inductively from the data. Data were managed, analyzed and coded by junior (LK, TF) and senior researchers (RPD, MK) of the study team (with backgrounds in Public Health and Health Services Research) using qualitative data analysis software (MAXQDA, 2018.2). Data coding and analysis were discussed continuously among the researchers (RPD, LK, MK and RPD, TF) to ensure intercoder congruity and to achieve the widest consensus possible. All researchers involved had prior experiences with qualitative methods. The pre-defined categories of the TDF were used to identify determinants of practice which influence healthcare delivery to patients with acute respiratory infections in general practices with regards to the educational components of the intervention and with regards to converting patterns of antibiotic use. Figure 3 describes the methodical approach.

## Results

### Socio-demographic characteristics

In the survey study sample, GPs were predominantly male (58% IG; 64% CG), with a mean age of 53 years and 25 years of professional experience. MAs had a mean age of 41 years and almost exclusively were female (100% IG; 98% CG). Across groups, MAs had been working for more than 15 years for the respective practice. 68% of participating GPs in the intervention group and 82% in the control group stated to have implemented changes in their practice in the last two years. GPs of the intervention group further stated to have visited a mean of 8.6 professional medical education workshops in the last six months. Table 2 gives an overview of socio-demographic characteristics of the quantitative study sample.

**Table 2:** Socio-demographics of survey participants (T1 *n* = 185; T2 *n* = 127)

	Intervention Group		Control Group	
T1	GP (*n* = 41)	MA (*n* = 50)	GP (*n* = 39)	MA (*n* = 55)
Age in years, mean (SD)	53 (9)	41 (12)	53 (10)	42 (12)
Sex f (%)	17 (42)	50 (100)	14 (36)	54 (98)
Experience years mean (SD)	25 (10)	17 (11)	23 (9)	17 (11)
Changes implemented in last 2 years yes (%)	28 (68)	34 (75)	34 (82)	38 (70)
**T2**	GP (*n* = 32)	MA (*n* = 32)	GP (*n* = 31)	MA (n = 32)
Age mean (SD)	54 (10)	41 (12)	57 (9)	41 (11)
Sex f (%)	13 (41)	32 (100)	9 (29)	32 (100)
Experience years in current role mean (SD)	25 (10)	17 (12)	27 (9)	16 (9)
Medical education workshops in the last six months mean (SD)	9 (6)	N/A	N/A	N/A

In total, 47 interviews were conducted between January and November 2019. Interviewed GPs (*n* = 16) had a mean age of 53 years, 24 years of professional experience and a nearly equal distribution of gender (region MV *n* = 10). Interviewed MAs (*n* = 7) had a mean age of 48 years, had worked with their employer for 16.7 years and were exclusively female (region MV n = 4). Patients (*n* = 16) had a mean age of 36 years and had been consulting the respective GP for eight years on average (region MV *n* = 11). To support anonymity of the small sample of OVs (n = 5) and GPs (*n* = 3) who participated in an in-depth interview regarding outreach visits, the only characteristic collected was related to gender. Table 3 summarizes the socio-demographic characteristics of the qualitative study sample.

**Table 3:** Socio-demographic characteristics of interview participants (*n* = 47)

Characteristics	GPs (n = 16)	MAs (*n* = 7)	Patients (n = 16)	OVs (*n* = 5) + VGPs^a^ (n = 3)
Age in years mean (SD)	53 (±8.29)	48 (±11.8)	36 (±12.2)	N/A
Sex f (%)	9 (56)	7 (100)	10 (62.5)	5 (62.5)
Expert years (mean SD)	24 (±8.2)	22 (±11.8)	N/A	N/A
Years with current employer mean (SD)	N/A	16.7 (±6.1)	N/A	N/A
Consulting this GP for years mean (SD)	N/A	N/A	8 (±8)	N/A

^a^*VGPS* GPs who had received an outreach visit

### Survey study

Survey waves were April to July (T1) and March to April 2020 (T2). A total of 109 practices (BW *n* = 54, MV *n* = 55) with 132 GPs were invited to participate in the survey. In T1, 185 participants responded of which 41 GPs (response rate 64%) and 50 MAs belonged to the intervention group. In the control group, 39 GPs (response rate 57.3%) and 55 MAs responded. Of these participants, 127 also answered the T2 survey questionnaire which represents a response rate of 69% across both participant groups. Here 32 GPs and 32 MAs in the intervention group and 31 GPs and 32 MAs in the control group returned the questionnaire until the set due date in April 2020. Five questionnaires were returned after the due date and could not be included for analysis. Overall, 312 survey questionnaires were included for analysis (response rate 58.3%).

Table 4 describes the uptake of all intervention components offered to the intervention group and the control group. Across time points, groups and occupational profession, the uptake of the website was between 40 and 50%. Here, uptake is defined by using the website as a counseling tool in order to strengthen patients’ health literacy competencies, however, it was also intended to strengthen healthcare professionals’ competencies. All paper-based material was provided to the practices in the intervention group. Control group practices received the mailing and could order their choice of material via the study-specific website. Initial uptake of the paper-based educational material was high. While the utilization of these components remained stable in the control group, it dropped in the intervention group over time. In the intervention group, adoption of the non-digital components by the participating MAs dropped from 86% in T1 to 41% inT2.

**Table 4:** Uptake of study components across study groups for T1 and T2

	Intervention Group		Control Group	
T1	GP (*n* = 41)	MA (*n* = 50)	GP (*n* = 39)	MA (*n* = 55)
Mail n (%)	33 (81)	45 (90)	31 (80)	42 (76)
Website n (%)	20 (49)	22 (44)	19 (49)	23 (42)
Information material n (%)	34 (83)	43 (86)	25 (64)	44 (80)
First feedback report n (%)	36 (88)	35 (70)	N/A	N/A
Outreach Visit n (%)	24 (59)	20 (40)	N/A	N/A
E-learning for teams n (%)	8 (20)	11 (22)	N/A	N/A
Tablet n (%)	18 (44)	29 (58)	N/A	N/A
**T2**	GP (n = 32)	MA (*n* = 32)	GP (n = 31)	MA (n = 32)
Mail n (%)	20 (63)	26 (81)	29 (93)	25 (78)
Website n (%)	14 (44)	13 (41)	N/A	13 (41)
Information material n (%)	16 (50)	13 (41)	23 (74)	24 (75)
Second feedback report n (%)	20 (63)	18 (56)	N/A	N/A
Outreach Visit n (%)	18 (57)	22 (69)	N/A	N/A
E-learning for teams n (%)	10 (31)	2 (6)	N/A	N/A
Tablet n (%)	15 (47)	20 (63)	N/A	N/A

The uptake of the intervention component feedback report dropped over the period of time. In T1, 88% of GPs used the first feedback report and considered it to be helpful in evaluating the performance of their practice. This number decreased to 56% in T2 for the second report. GPs and MAs rated the outreach visit to be beneficial in T1 and T2. The e-learning platform had the lowest rate of adoption compared to all intervention components. In total, only 10 GPs and 11 MAs reported using this format which could be reached via personalized access. Approximately half of respondents offered the tablet devices to patients. Noticeably, the uptake dropped over time whereby MAs appeared to have a higher rate of adoption compared to GPs.

In addition to the general uptake of study components, Table 5 illustrates the extent of the concordant uptake between GPs and MAs working in one facility. This overlap is shown for the intervention group only since particular interest was on intervention components. In general, the overlap of the digital intervention components (tablet; e-learning platform) was considerably high. In T1, 83% of responding practices were consistent in the uptake of tablet devices in their practice team. In T2, this consistency slightly decreased to 65%. Considering absolute numbers, the consistency in the perception of outreach visits stayed stable over time. In comparison, the perception of feedback reports providing helpful information for performance evaluation of the practice dropped from 76 to 65%.

**Table 5:** Overlap of self-reported component uptake within practices

Device	T1 (practices n = 41)	T2 (practices *n* = 31^a^)
Mailing n (%)	30 (73.2)	25 (80.6)
Website n (%)	25 (61)	20 (64.5)
Feedback n (%)	31 (75.6)	20 (64.5)
Outreach visit n (%)	23 (56.1)	21 (67.7)
E-learning n (%)	34 (82.9)	25 (80.6)
Tablet n (%)	34 (82.9)	20 (64.5)
Flyer	N/A	27 (87.1)
Coloring book	N/A	17 (54.8)
Plush Toy^b^	N/A	17 (54.8)
COLD magazine^b^	N/A	26 (83.9)
Comic^b^	N/A	19 (61.3)
Printed and online information for professionals	N/A	18 (58.1)

^a^ questionnaires returned by GPs and MAs from 31 practices

^b^Only offered in second half of the intervention period

### Interview study

Between January and April 2019, semi-structured guide-based telephone interviews were carried out with a sample of intervention group GPs (n = 16), MAs (n = 7), and patients (n = 16) from the intervention group and the control group with a mean duration of 22.1 min. In addition, semi-structured guide-based interviews were conducted in October and November 2019 with 4 outreach visitors, 1 expert and three GPs who had received an outreach visit to broaden obtained findings regarding the visits. Here, the mean duration was 27.5 min.

All interviews added in-depth understanding of the educational intervention components, context factors impacting the uptake of the intervention and the significance and role of the implemented set of measures to practice teams and patients. Out of 14 deductively applied TDF domains, findings are reported in relation to three TDF constructs that emerged as main categories from the analysis: Environmental context and resources; Social/professional role and identity; Beliefs about consequences. Other TDF constructs such as Emotion, Skills and Optimism were less prevalent. Since a utilization gap between digital and paper-based study components was identified post hoc, these component groups as well as the outreach visits are reflected. Included quotes have been translated into English with due diligence and are cited with transcript position.

### Environmental context and resources

#### Outreach visits

A total of 44 on-site outreach visits and 4 telephone visits were conducted. In general, on-site visits were attended by GPs and MAs, in some cases by the GP only. The telephone visits were conducted on individual request with the respective GP. Due to refusal or drop-out, 9 visits could not take place. OVs reported that they were met with reluctance and had to overcome difficulties in scheduling of the visits already. Efforts to reach practices in rural areas were considered too high and premises often inadequate. In general, OVs were made aware of the scarcely available resource of time in practices which was repeated when they arrived for the outreach visit: *“now in fast forward, we don’t have time”* (OV3, 01:53). On practice site, the re-occurring issue of sub-optimal premises restricted the options for a set-up in a quiet location to deliver the prepared presentation and materials and to discuss the feedback report. In some cases, this meant to deliver the intervention component during lunch hours and in break rooms *“between the garbage bin and the coffee maker”* (VExp; 23:18). In a more figurative sense and emotionally shaped perspective on the environment, OVs felt that the visits and they themselves were seen as disruptive for routine processes. Moreover, they experienced GPs to play down the relevance of visits since elements referring to participatory communication were not seen as crucial and available data presented in feedback reports was not seen as a significant resource: *“Not all of them thought it was interesting, there were critical voices, too, data too old, nothing in it, we don’t prescribe antibiotics at all* “(OV01, 08:47). In contrast to OVs’ statements, interviewed GPs saw the visit as a beneficial resource and felt motivated by the comparison of prescription rates between practices. OVs mentioned that this benchmarking supported them in conveying improvement potential in prescribing patterns, particularly if GPs initially perceived their prescription rates as flawless. According to the OVs, the outreach visits could not be delivered as planned and according to the developed concept they were made familiar with since diverse contextual factors required a certain extent of flexibility, tailoring and deviation from the implementation plan.

*“You have to deliver [the intervention] in a way people can accept it”* (OV02, 03:21).

*“In other words, the intervention never was delivered in a standardized way. … practice did not expect a presentation, … , suitable room not available, improvised in the consulting room, contents of the feedback report not known, but interested in topic.”* (OV4; 03:08–04:53)

*„comparing to other practices was very helpful, I had the impression that this combination - create awareness and provide information - … actually a good thing, because it opens doors“. (OV02, 04:43)*

#### Non-digital intervention components

When the interviews were conducted, rolled out non-digital intervention components were still limited to paper-based flyers and posters containing educational relevant information. Consistent with survey findings, the uptake of these two components was reported to be high. Especially MAs saw benefits in providing patients with information material which was perceived to be appealing. Flyers in particular had a high rate of adoption which was explained by a suitable integration into practice routines. *“Well, I have to say the flyers, they yield a lot, because people actually engage with them”* (M4; 10:24). GPs used flyer in counselling situations and considered them fitting to give to patients instead of a prescription for antibiotics. *“People want to hold something in their hands when they leave the practice, if not a prescription for antibiotics then a recommendation how to inhale and drink different tees”* (GP10; 12:23).

#### Digital intervention components

The public campaign which included a website met challenges in reaching the target group. Patients could not clearly differentiate the campaign from other sources of information. One patient even wrongly attributed a televised spot to the campaign. Nevertheless, patients hypothesized that a reliable and trustworthy web-based source of information would be helpful in creating thematic awareness and increasing health literacy and considered the GP practice to be a suitable location to learn about such a website.

While no interviewed MAs had used the e-learning module, two interviewed GPs stated to have done so and considered it helpful for handling patients’ expectations. In particular, they expected benefits for situations in which patients actively requested antibiotics and thought that communicative elements conveyed by the module could help them to promote alternative treatment approaches: *“Yes, very artificial scene, but I thought it was good to create awareness [for patient expectations]”* (GP6; 06:58). GPs who had not used the e-learning module reported they had not been aware of its existence.

During the period of conducting interviews, tablet devices had only been available to the practices for a short time. In a few practices, the tablet had not been available yet or was refused completely. Opinions about using tablet devices for information provision in GP practices were heterogenous. Critics argued that only one person at a time could use a tablet. Additionally, hygiene issues were discussed as major concerns and grounds for potential refusal. Additional stress and resource-consuming efforts for the team were anticipated regarding dispensing and monitoring whereabouts of the tablets. Supporters of tablet devices saw opportunities to extend application areas: In order to avoid uncomfortable situations with GPs, they could help to address sensitive topics. Due to prioritizing consultation topics in advance, this would also lead to an intensified utilization of consultation time. The interviewed patients were not aware of the tablet devices, but reflected on a potential use. Waiting room TVs were proposed as a proper alternative for digital information provision. GPs, MAs and patients shared concerns regarding the uptake of tablet devices and also voiced a general fear of theft. From their perspective, a standardized procedure would be required to define when and how to let patients use the tablet and to take a deposit of some sort to ensure it would be returned again.

*“I think perhaps you would have to have several [tablets]. Well, I think a screen everybody can look at would be simpler. Or, 15 to 20 tablets would be necessary so everybody [in the waiting area] could use them.” (P10; 18:53)*

*“Tablets are an option that would be very appealing to me. Then you don’t have to ask the GP again, which could make others [patient] uncomfortable.” (P14: 8:45-9:22)*

### Social/professional role and identity

#### Outreach visits

OVs experienced a diverse integration of MAs in visits. Predominantly, the whole practice team was attending, but in some cases, GPs denied the participation of MAs. Here, OVs presumed that GPs did not want to create an additional burden for MAs since the GPs themselves are responsible for antibiotic prescription rates. In other cases, MAs participated in the visit while the GP refused to do so. One interviewed GP saw the visit as opportunity to foster team building and considered reflection on internal routines more suitable with an external visitor than within the team only. GPs who favored homeopathy and naturopathic medicine conveyed that in their understanding, other medical specialist groups such as pediatricians would be responsible for high antibiotics prescription rates in primary care, not they themselves. With regard to intervention fidelity, OVs felt that practices with background in academic teaching demonstrated stronger adherence to the protocol.

*“It was very good [the visit]. I have 4 MAs and it is always beneficial when you are not he only one conveying knowledge, but to have someone external coming in and talk about antibiotic resistances.” (VGP03; 11:30)*

*“… I visited two or three practices … that we have worked with before and over years. You noticed that everything ran differently there. These are academic teaching practices and they took more time.” (OV1; 18:42)*

#### Non-digital components

Paper-based components appeared to be in line with the perceived social professional role of interviewed GPs and MAs and were considered to be supportive for their daily routines. GPs generally supported the idea to increase health literacy competencies in patients. Thus, poster and flyer were seen as constant reminders for the appropriate use of antibiotics in acute respiratory tract infections. Patients contemplated their willingness to engage with educative health-related information in medical practices. It was assumed that in case of consulting a GP for an acute respiratory infection, they might lack receptivity while sitting in a waiting area and prefer to be educated by the GP directly.

*“… I believe, this is the main factor, you actually know it somehow, but often you don’t think of it anymore, right? Such a campaign supports simply thinking of it and being aware.” (GP7; 09:58)*

*“I don’t know. When I am sick, then I am really sick and when I sit there, then I personally have no interest in [ … ] then I am happy to see the GP quickly and get out again fast.” (P17; 10:56-11:12)*

#### Digital components

In contrast to paper-based components, the acceptance of tablet devices was mixed. Refusing GPs saw their practice as a place of tranquility and protection. Thus, in days of constant information flooding and availability, they consciously wanted to establish a “safe harbor” for patients. Following this perception of the social professional role of a GP practice, tablet devices were seen as a disruption, neither fitting the needs of patients nor GPs or the team. To some extent, MAs seemed to adopt their employers’ positions.

Interviewed GPs and MAs were not familiar with the study-specific website. At the time of the interviews, less than half of the interviewed professionals had visited the platform, yet they generally supported the idea of providing a site with evidence-based reliable information. GPs who used the e-learning tool appreciated the input of communicative training elements in which they gathered ideas on how to respond to irrational patient demands for antibiotic therapy. Patients saw a general need for trustworthy sources of health-related information, but had not come across the study-specific website.

*“I think people already are getting bombarded enough with this stuff. I don't want that in the practice here. We actually also have a ban on mobile phones here. So, of course you can play around on your mobile phone, but you must not talk on the phone [ …] I believe that this is also quite good for the patients if they come to rest for a few minutes in the waiting area. In this respect, I don't see a place for it in my practice now.” (GP14; 18:10)*

### Beliefs about consequences

#### Outreach visits

As applied and in retrospect, OVs and the interviewed expert did not consider the visits to be a suitable intervention component for the CHANGE-3 study. GPs argued that feedback reports on their antibiotics prescribing were of minor relevance to them since case numbers appeared to be small and lacked contextual interpretation. To accommodate scheduling issues, OVs conducted a total of 4 visits via telephone which was considered to have worked surprisingly well. To increase GPs’ motivation to participate in visits, OVs suggested offering a higher compensation. Even though experiences of OVs about visits seemed to be connected with defiance, interviewed GPs considered the visits to be beneficial. They acknowledged the included feedback reports which offered rational benchmarking and saw ways to include their staff into sustainable care quality improvement mechanisms.

*„ … using [outreach visits] widely would surely be extremely complex and complicated. So, for sure it was good to test it again for such a relevant topic, but I believe it probably cannot be used in routine care, because it is relatively expensive … I am afraid this concept would rather be suitable for other things” (VExp; 17:34*)

#### Non-digital components

The most significant consequence of paper-based components was seen in an increased awareness of an appropriate use of antibiotics. For GPs, the daily confrontation with poster and flyer worked as a constant reminder to question if antibiotics were indicated. For MAs, the material assisted to meet the patients’ needs in providing them information they could rely on and which guided them in handling their infection.

### Digital components

Referring to the public campaign, chances were recognized for providing a reliable source of relevant information via a trustworthy website. As awareness of the study-specific website was limited, statements and beliefs were primarily hypothetical considerations.

*“Well, that would tie up far too much time at the front desk, if a MA first has to explain what to do with the tablet, then she has to collect a deposit for it and then return it, disinfect it, that would tie up far too much MA working time, so we didn't use it.” (GP04; 06:54)*

*“Yes, and something has been stolen before, somehow a painting even from the wall, and we just didn't want to induce this stress with this tablet now, [...] we had this conversation in the practice [ … ]” (M01; 12:03)*

## Discussion

Based on selected TDF domains, the systematic analysis in this process evaluation shows a high consistency in the uptake of non-digital study components between qualitative and quantitative data, with the exception of a data inconsistency in the uptake of website and tablets. The reported findings suggest apparent differences referring to the adoption of paper-based and digital intervention components related to the mode of delivery, not to the delivered intervention component itself. Non-digital components suited needs and preferences of GPs and MAs which was mainly attributed to an effortless adoption into daily routines. Digital components - and tablet devices in particular - were not considered to easily fit into practice routines and care processes. Where GPs felt that implementing them into care routines would contradict their own belief system, they actively engaged in keeping their practice a place protected of excessive digital supply and excluded respective intervention components to provide a place of tranquility to their patients.

A recent systematic review [27] identified barriers and facilitators in the implementation of e-health devices. Main barriers were concerns about theft, absence of motivation to use offered devices, a perceived added workload and general issues in adopting e-health devices into organizational routines. Among found facilitators were an ease of use, staff motivation, involvement of all stakeholders and the availability of necessary resources. The findings in this PE seem to match these results, but also indicate that motivation to routinely apply the delivered components was not strong. A lack of motivation could also have contributed to the low uptake of the e-learning module.

The educational website for the regional campaign was considered to be visually appealing by participants, yet it lacked in awareness and targeted dissemination strategies. Detailed results regarding the application of digital information delivery in the CHANGE-3 study have been reported [28]. Findings might be very specific for the current older generation of GPs and patients in Germany, or even with regards to gender, particularly as they partially confirm results of prior research in German primary care: A randomized controlled trial concluded that such findings needed to be replicated and that - although information technology can support effective interventions - disadvantages such as technical requirements of the users and devices should also be considered when integrating digital solutions into wider care-related situations and populations [29]. Also, a study investigating learning, training and use of information technology among GP trainees aged 30 to 50 showed a high variation regarding affinity and concluded that there is a need for support and better understanding of information technology systems among primary care physicians [30]. However, findings of this PE point to a need for a more precise fit to practice routines so digital intervention components will not be regarded a burden.

Interactive and personal educational outreach visits are considered to be effective [21] and can have relatively consistent and small, but potentially important effects on prescribing patterns when used alone or combined with other interventions [22]. However, OVs in this study faced diverse obstacles in their efforts to apply visits as planned. Organizational challenges forced them to tailor visits and deviate from the implementation plan as GPs only reluctantly acknowledged visits and feedback reports. Their main criticism referred to relevance of the data for their current situation or a general doubt of the appropriateness which led to rejection. This correlates with findings of prior research [31] which identified that GPs compared to surgeons only saw a low relevance of antibiotic resistances for their daily work (32% vs 82% in surgeons). Similar to this PE, they also found that GPs tended to assume that other medical specialist groups were responsible for high antibiotic prescription rates in primary care, not the GPs themselves.

The small number of outreach visits conducted over telephone points to the potential for delivering this intervention component in a cost- and more time-effective manner. Also, video formats could serve as a useful option, but possibly cannot replace face-to-face visits as an outreach visit model [32]. Nevertheless, formats that use web conference tools might pose a viable alternative, particularly as they currently gain importance and meet a high degree of acceptance and usage in times of Covid-19.

A review evaluated effects of public campaigns aiming at improving the use of antibiotics in respiratory tract infections. It was found that awareness problems occurred in several studies using public campaigns, yet results suggested positive effects on the use of antibiotics and that multifaceted campaigns repeated over several years might have the greatest benefits [33]. Confirming these results, awareness of the study-specific website in CHANGE-3 was low. Future pragmatic trials with public campaign initiatives therefore should carefully consider the potential for dissemination in a multifaceted campaign and aim for long running and also repeated approaches.

The CHANGE-3 study was one of a number of efforts to change antibiotics use patterns in German primary care conducted in recent years. Other studies focused on knowledge about factors causing antimicrobial resistance and strategies fostering a decline in prescription rates of antibiotics like delayed prescribing [34], online education on provider-patient-communication [35], attitudes and perceptions to antimicrobial resistances and preferred information sources [31] or used complex implementation programs to promote the appropriate use of antibiotics in primary care networks [36]. Impulses for primary care gained in these studies were collated in a recent symposium [37]. The implementation program in CHANGE-3 used an innovative component to contribute to a further development of complex interventions in this research field: A study-specific website based on knowledge translation strategies provided evidence-based information about the use of antibiotics to the public and to health professionals at the same time. This approach was combined with intervention components which focused on reflection of own habitual patterns in practice teams and provider-patient communication. Applying selected domains of the TDF facilitated insights into key actor behavior patterns and the identification of barriers for the implementation program. Using the TDF in the evaluation of a complex intervention supported a comprehensive view on cognitive, affective, social and environmental determinants of patterns of antibiotics use and represents a novel contribution in German primary care.

### Strengths and limitations

The results of the PE strengthen the reporting in CHANGE-3 and provide information for future implementation programs. Analysis of all data was guided by adequate methodological strategies and aimed at minimization of bias and a reduction of the risk of losing relevant content. Reporting of the completed process evaluation findings follows the recommendations of the COREQ checklist (COnsolidated criteria for REporting Qualitative research) [38] and the Standards for Reporting Implementation Studies (StaRI) Statement [39] and adheres to the CONSORT guidelines [40]. Data triangulation occurred through an integrated approach of systematic incorporation of qualitative and quantitative data after individual analysis of all collected data. This approach facilitated a more holistic view and profound description of results. The interview study provided additional insights into the reach and fidelity of the implementation program and perceived effects of the educational intervention components on the daily practice of healthcare delivery to patients with ARTI. Telephone interviews ensured that the additional burden for participants was minimized to the maximum extent. Using a conceptional framework approach ensured considerations of theory driven key themes. This procedure was combined with an inductive approach to identify additional themes. In the process of analysis, a high inter-coder congruence was achieved which reflects a reliable classification of data. Since all interested parties could be included in the interview study, the relatively high number of interviews allowed for a thorough analysis. It provided sufficient data to illustrate pre-determined theoretical as well as well as inductive categories. All interview data were analyzed until no new themes emerged and identification of deviant observations and consistency of findings facilitated the assessment of data sufficiency and thematic saturation.

Limitations are apparent in the small quantitative sample where ratios in the uptake of study components pose the risk of overinterpretation of results. Values are exclusively descriptive which prevents interpretation of significant changes in time or across groups. Some practices also had participated in a prior study with similar objectives which allows for a possible interfusion of potential effects. All findings need to be considered cautiously since it can be assumed that participating GPs already had been engaging in efforts that aimed to convert habitual patterns regarding the use of antibiotics for ARTI and thus their MAs also had been exposed to the topic in more detail prior to the study. Analyses performed to investigate claims-data-based outcomes are still pending, but will be used to contextualize the findings of the process evaluation.

## Conclusion

Domains derived from the TDF supported the exploration and understanding of facilitators and barriers to the implementation strategy in CHANGE-3. Thus, this PE contributed to further assessment of the applicability of the educational components delivered to primary care settings and the general public in Germany with regards to the appropriate use of antibiotics. Future research efforts need to focus on the mode of delivery, not to the delivered intervention component itself to reach the targeted population.

## Supplementary Information

**Additional file 1.** Interview guide (general practitioners; translated version).

**Additional file 2.** Interview guide MAs (translated).

**Additional file 3.** Interview guide patients (translated).

**Additional file 4.** Interview guides referring to outreach visits (translated version).

**Additional file 5.** Survey questionnaire T1 – GPs.

**Additional file 6.** Survey questionnaire T1 – MAs.

**Additional file 7.** Survey T2 GPs translated.

**Additional file 8.** Survey T2 MAs translated.

## Data Availability

All data generated and analyzed during this PE are stored on a secure server at the University Hospital Heidelberg, Germany, Department of General Practice and Health Services Research. De-identified sets of the data collected and analyzed during this study can be made available by the corresponding author on reasonable request.

## Abbreviations

AOK: Allgemeine Ortskrankenkasse (statutory health insurer)
ARTI: Acute Respiratory Tract Infections
aQua: Institute of applied quality improvement and research in health care
BW: Baden-Wuerttemberg
BMG: Federal Ministry of Health (Bundesgesundheitsministerium)
CHANGE-3: Converting Habits of Antibiotic Use for Respiratory Tract Infections in German Primary Care
CG: Control Group
GP: General Practitioner
IG: Intervention Group
MA: Medical Assistant
MV: Mecklenburg-Vorpommern
OV: Outreach visitor
P: Patient
PE: Process Evaluation
SD: Standard Deviation
VExp: Outreach visit expert
VGP: GP who received an outreach visit
