# Supplementary material for: Converting habits of antibiotic use for respiratory tract infections in German primary care (CHANGE-3) - process evaluation of a complex intervention
Source: BMC Fam Pract. 2020 Dec 19;21:274. doi: 10.1186/s12875-020-01351-2 (PMC7749701; doi:10.1186/s12875-020-01351-2)
Supplement: Supplementary file 1 — Additional file 1. Interview guide (general practitioners; translated version). [file 12875_2020_1351_MOESM1_ESM.docx]

**Additional file 1: Interview guide (general practitioners; translated version)**

**A – Exposure to antibiotics**

Please describe your regular course of action when taking therapy decisions for/against antibiotics regarding patients with acute respiratory tract infections

- Influence of patient preferences
- Methods or strategies in use (delayed prescribing?)

**B – Uptake of offered intervention components**

Which of the CHANGE-3 intervention components were participants aware of?

- Why? / Reasons?
- Components not used / Why?

Transfer of information provided during outreach visit and feedback report into daily routines?

- How informative?
- Resources needed to implement the recommendations into daily routines?

Role of utilized intervention components in future decision-making processes regarding patients with acute respiratory tract infections?

- Change of attitude
- Transfer to decision-making process regarding other diseases (e.g. urinary tract infection)

**C – Dissemination of key messages**

Next to interventions addressing the practice level, a broad public campaign with information material (poster, website, colouring books, comics) has been out rolled for the public and the professional audience. How could you pass on the related information to patients with acute respiratory tract infections?

- Aspects of campaign considered positive /helpful

Which intervention component supported you and your team in passing on this message?

Changes observed in patients?

- Patient expectations
- Relevant differences

**D – Conclusion**

Comments and ideas regarding the development of the project?

- Recommendations for future usage of antibiotics for acute respiratory infections

What would you like to tell us besides already discussed topics?
