# Supplementary material for: Converting habits of antibiotic use for respiratory tract infections in German primary care (CHANGE-3) - process evaluation of a complex intervention
Source: BMC Fam Pract. 2020 Dec 19;21:274. doi: 10.1186/s12875-020-01351-2 (PMC7749701; doi:10.1186/s12875-020-01351-2)
Supplement: Supplementary file 2 — Additional file 2. Interview guide MAs (translated). [file 12875_2020_1351_MOESM2_ESM.docx]

**Additional file 2: Interview guide MAs (translated)**

**A – Exposure to antibiotics**

Please describe your range of tasks regarding patient care in your practice.

- Role of counselling

Which experiences did you make in dealing with patients with acute uncomplicated infections?

- Personal approach
- Communication with patients
- Strategies (recall system)

**B – Uptake and impact of offered intervention components**

Which of the intervention components do you consider helpful in treating patients with acute respiratory tract infections?

- Why / Explanation
- Adaption to personal work range
- Uptake of components / challenges regarding components

To what extent did the components support your daily work in the practice and in care of patients with acute respiratory tract infections?

- Change of range of tasks
- Change of patient interaction
- Change of communication in practice team

Personal perception of knowledge regarding antibiotics after implementing the intervention components?

- Updated knowledge / newly gained knowledge

**C – Dissemination of key messages**

Support by outreach visit and feedback report to bring campaign material closer to patients?

- Elements of public campaign considered important / less important
- Role of antibiotics resistance in daily work

Patient reactions observed?

- Patient expectations
- Organisational factors / communication in team / team structure

**D – Conclusion**

Which ideas and comments do you have for a further development of the interventions you got to know in the study?

- Recommendations for usage of antibiotics in patients with acute respiratory tract infections

What would you like to tell us besides already discussed topics?
