# Supplementary material for: Converting habits of antibiotic use for respiratory tract infections in German primary care (CHANGE-3) - process evaluation of a complex intervention
Source: BMC Fam Pract. 2020 Dec 19;21:274. doi: 10.1186/s12875-020-01351-2 (PMC7749701; doi:10.1186/s12875-020-01351-2)
Supplement: Supplementary file 4 — Additional file 4. Interview guides referring to outreach visits (translated version). [file 12875_2020_1351_MOESM4_ESM.docx]

### Additional file 4: Interview guides referring to outreach visits (translated version)

### Interview guide for expert

A: Aim of outreach visits

What are the basic ideas underlying the practice visits within this trial?

What was their purpose within the trial?

B: Conception and conduction of outreach visits

Please describe the process of conception of the visits.

What was the base of conception?

How were visitors prepared for the visits? How were they selected?

Please describe the process of an ideal outreach visit.

What are the pitfalls of successful conduction in your opinion?

C: Evaluation of practice visits

How do you evaluate the success of the conducted outreach visits?

If you were to design outreach visits again – what would you do differently?

### Interview guide for outreach visitors

A: Please describe how you typically conducted the outreach visits.

How did you prepare for the practice visits?
Which materials or media were used?

B: Perception of visits

In your estimation, how did the practice teams perceive the visits?

How did they cope with the visits?

Which obstacles did you encounter? How did you deal with them?

How do you evaluate the benefit for the visited practices?
How do you determine that?

If you had to do a visit again today, what would you do differently?

What else would you like to mention regarding the outreach visits?

What skill set does a visitor need to successfully conduct visits?

Where else could practice visits be used?

### Interview guide for GPs

A: Please describe the outreach visit you received in your practice.
What about scheduling and organization of the visit?

Who was present?

Which materials did you receive during the visit?

What do you think about alternative formats?

B: Perceptions

What do you generally think about outreach visits?

What did you like about the visit? What did you not like?

Where did you perceive difficulties?

How should a visit ideally be done?

What was the benefit of the visit for your practice? What could you draw from it for your daily work? How did you establish the benefit?

How could the process of an outreach visit be optimized?

What else would you like to mention concerning the outreach visit?
