# Supplementary material for: Converting habits of antibiotic use for respiratory tract infections in German primary care (CHANGE-3) - process evaluation of a complex intervention
Source: BMC Fam Pract. 2020 Dec 19;21:274. doi: 10.1186/s12875-020-01351-2 (PMC7749701; doi:10.1186/s12875-020-01351-2)
Supplement: Supplementary file 5 — Additional file 5. Survey questionnaire T1 – GPs. [file 12875_2020_1351_MOESM5_ESM.docx]

**Additional file 5: Survey questionnaire T1 – GPs**

| **The study-specific homepage** [**www.weniger-antibiotika.de**](http://www.weniger-antibiotika.de) **(less-antibiotics)** | **Disagree strongly** | **Disagree** | **Neutral** | | **Agree** | **Agree strongly** |
| --- | --- | --- | --- | --- | --- | --- |
| … was visited by me |  |  |  | |  |  |
| … contains current information new to me |  |  |  | |  |  |
| … informs me in a comprehensible manner |  |  |  | |  |  |
| … strengthened me in dealing with patient expectations |  |  |  | |  |  |
| … I considered helpful for patient interaction |  |  |  | |  |  |
| … I recommended to my patients |  |  |  | |  |  |
| … strengthens my decision for or against prescribing antibiotics |  |  |  | |  |  |
| … has an impact on my interaction with patients |  |  |  | |  |  |
| … has an impact on the therapy patients with acute respiratory tract infections receive |  |  |  | |  |  |
| … lead to a decline in antibiotics prescriptions |  |  |  | |  |  |
| **The offered e-learning platform** | **Disagree strongly** | **Disagree** | | **Neutral** | **Agree** | **Agree strongly** |
| … was used by me |  |  | |  |  |  |
| … contains current information new to me |  |  | |  |  |  |
| … informs me in a comprehensible manner |  |  | |  |  |  |
| … strengthened me in dealing with patient expectations |  |  | |  |  |  |
| … I considered helpful for the patient interaction |  |  | |  |  |  |
| … motivates to follow a guideline-based treatment |  |  | |  |  |  |
| … strengthens my decision for or against the prescription of antibiotics |  |  | |  |  |  |
| … has an impact on my interaction with patients |  |  | |  |  |  |
| … has an impact on the therapy patients with acute respiratory tract infections receive |  |  | |  |  |  |
| … leaded to a decline in the prescription of antibiotics |  |  | |  |  |  |
| **The tablet with relevant information for patients** | **Disagree strongly** | **Disagree** | | **Neutral** | **Agree** | **Agree strongly** |
| … is available in the practice |  |  | |  |  |  |
| … is used by my patients |  |  | |  |  |  |
| … is helpful to discuss treatment options with patients |  |  | |  |  |  |
| … strengthened me in dealing with patient expectations |  |  | |  |  |  |
| … motivates to follow a guideline-based treatment |  |  | |  |  |  |
| … is relieving my decision for or against a antibiotics-therapy |  |  | |  |  |  |
| … gives me confidence in dealing with patients’ expectations |  |  | |  |  |  |
| … has an impact on my interaction with patients |  |  | |  |  |  |
| … has an impact on the therapy patients with acute respiratory tract infections receive |  |  | |  |  |  |
| … lead to a decline in the prescription of antibiotics |  |  | |  |  |  |
