# Supplementary material for: Converting habits of antibiotic use for respiratory tract infections in German primary care (CHANGE-3) - process evaluation of a complex intervention
Source: BMC Fam Pract. 2020 Dec 19;21:274. doi: 10.1186/s12875-020-01351-2 (PMC7749701; doi:10.1186/s12875-020-01351-2)
Supplement: Supplementary file 6 — Additional file 6. Survey questionnaire T1 – MAs. [file 12875_2020_1351_MOESM6_ESM.docx]

**Additional file 6: Survey questionnaire T1 – MAs**

| **The study-specific homepage** [**www.weniger-antibiotika.de**](http://www.weniger-antibiotika.de) **(less-antibiotics)** | **Disagree strongly** | **Disagree** | **Neutral** | **Agree** | **Agree strongly** |
| --- | --- | --- | --- | --- | --- |
| … was visited by me |  |  |  |  |  |
| … contains current information new to me |  |  |  |  |  |
| … informs me in a comprehensible manner |  |  |  |  |  |
| … is helpful in communication with the patients |  |  |  |  |  |
| … motivates me to support the GP even more intensively in the care of patients with acute respiratory tract infections |  |  |  |  |  |
| … strengthens the trust in my own competencies for an intense participation in patient-care |  |  |  |  |  |
| … gave me confidence for the communication with patients |  |  |  |  |  |
| … has an impact on the level of dedication I have in patient-care |  |  |  |  |  |
| **The offered e-learning platform** | **Disagree strongly** | **Disagree** | **Neutral** | **Agree** | **Agree strongly** |
| … was used by me |  |  |  |  |  |
| … contains current information new to me |  |  |  |  |  |
| … informs me in a comprehensible manner |  |  |  |  |  |
| … motivates me to support the GP even more intensively in the care of patients with acute respiratory tract infections |  |  |  |  |  |
| … strengthens the trust in my own competencies for an intense participation in patient care |  |  |  |  |  |
| … gave me confidence for the communication with patients |  |  |  |  |  |
| … has an impact on the level of dedication I have in patient-care |  |  |  |  |  |
| **The tablet with relevant information for patients** | **Disagree strongly** | **Disagree** | **Neutral** | **Agree** | **Agree strongly** |
| … is available in the practice |  |  |  |  |  |
| … is used by the patients |  |  |  |  |  |
| … is helpful for my daily work in the practice |  |  |  |  |  |
| … motivates me to get even more involved in the care of patients |  |  |  |  |  |
| … gives me confidence for the communication with patients |  |  |  |  |  |
| … gives me confidence in dealing with patients’ expectations |  |  |  |  |  |
| … has an impact on my interaction with patients |  |  |  |  |  |
| … influences the way I involve myself into the care of patients with acute respiratory tract infections |  |  |  |  |  |
