# Supplementary material for: Converting habits of antibiotic use for respiratory tract infections in German primary care (CHANGE-3) - process evaluation of a complex intervention
Source: BMC Fam Pract. 2020 Dec 19;21:274. doi: 10.1186/s12875-020-01351-2 (PMC7749701; doi:10.1186/s12875-020-01351-2)
Supplement: Supplementary file 7 — Additional file 7. Survey T2 GPs translated. [file 12875_2020_1351_MOESM7_ESM.docx]

**Additional file 7: Survey T2 GPs translated**

| **A. Interventions of the CHANGE-3-study** | | | | | | |  |
| --- | --- | --- | --- | --- | --- | --- | --- |
| **I used the following CHANGE-3 interventions and utilized them in practice:** | | | | | | | |
| 1) Mail  2) Mail for professional audience  3) Individualized data-based feedback report regarding antibiotic prescription  4) Outreach visit  5) Website (weniger-antibiotika.de)  6a) Patient information flyer regarding different acute, uncomplicated infections, German  6b) Patient information flyer regarding different acute, uncomplicated infections, foreign language | 7) E-learning training program regarding patient centred communication  8) Tablet PC containing patient information  9) Hatschi (colouring book)  10) Hatschi plush toy  11) COLD-magazine  12) Comic addressing school children | | | | | | |
| **The following CHANGE-3 interventions provided new impulses (input, knowledge, strategies) concerning the treatment of patients with acute, uncomplicated infections:** | | **Disagree**  **Strongly** | **Disagree** | **Neutral** | **Agree** | **Agree**  **Strongly** | |
| 1) Mail | |  |  |  |  |  | |
| 2) Mail for professional audience  (online and print) | |  |  |  |  |  | |
| 3) Individualized data-based feedback report regarding antibiotic prescription | |  |  |  |  |  | |
| 4) Outreach visit | |  |  |  |  |  | |
| 5) CHANGE-3-website (weniger-antibiotika.de) | |  |  |  |  |  | |
| 6a) Patient information flyer regarding different acute, uncomplicated infections, German | |  |  |  |  |  | |
| 6b) Patient information flyer regarding different acute, uncomplicated infections, foreign languages | |  |  |  |  |  | |
| 7) E-learning training program regarding patient centred communication | |  |  |  |  |  | |
| 8) Tablet PC containing patient information | |  |  |  |  |  | |
| 9) Hatschi (colouring book) | |  |  |  |  |  | |
| 10) Hatschi plush toy | |  |  |  |  |  | |
| 11) COLD-magazine | |  |  |  |  |  | |
| 12) Comic addressing school children | |  |  |  |  |  | |

| **A-1: The information material for the general public** | **Disagree**  **Strongly** | **Disagree** | **Neutral** | **Agree** | **Agree Strongly** |
| --- | --- | --- | --- | --- | --- |
| … was noticed by me |  |  |  |  |  |
| - *If you „disagree strongly“ please skip to* ***A-2*** |  |  |  |  |  |
| ... reach my patients |  |  |  |  |  |
| … I consider helpful for patient interaction |  |  |  |  |  |
| … facilitates my decision for or against the prescription of antibiotics |  |  |  |  |  |
| … strengthens me in dealing with patient expectations |  |  |  |  |  |
| … influence my patient interaction |  |  |  |  |  |
| … influence which therapy patients with acute respiratory tract infections receive. |  |  |  |  |  |

| **A-5: The tablet PC containing patient information** | **Disagree Strongly** | **Disagree** | **Neutral** | **Agree** | **Agree Strongly** |
| --- | --- | --- | --- | --- | --- |
| … is in use in my practice |  |  |  |  |  |
| - *If you „disagree strongly“ please skip to* ***B*** |  |  |  |  |  |
| … facilitates my decision for or against the prescription of antibiotics |  |  |  |  |  |
| … provides me safety in dealing with patient expectations |  |  |  |  |  |
| … influences my patient interaction |  |  |  |  |  |
| … has led to a decrease in antibiotic prescriptions. |  |  |  |  |  |

| **B-1: In general, the prescription of antibiotics in patients with acute respiratory tract infection leads to** | **Disagree Strongly** | **Disagree** | **Neutral** | **Agree** | **Agree Strongly** |
| --- | --- | --- | --- | --- | --- |
| ... the feeling of being a competent physician. |  |  |  |  |  |
| … the impression of being a competent physician in my peers’ eyes. |  |  |  |  |  |
| … a reduced consultation time |  |  |  |  |  |
| ... a lower frequency of consultations |  |  |  |  |  |
| … satisfied patients |  |  |  |  |  |
| … patient demands for antibiotics in future consultations. |  |  |  |  |  |
