# Supplementary material for: Converting habits of antibiotic use for respiratory tract infections in German primary care (CHANGE-3) - process evaluation of a complex intervention
Source: BMC Fam Pract. 2020 Dec 19;21:274. doi: 10.1186/s12875-020-01351-2 (PMC7749701; doi:10.1186/s12875-020-01351-2)
Supplement: Supplementary file 8 — Additional file 8. Survey T2 MAs translated. [file 12875_2020_1351_MOESM8_ESM.docx]

**Additional file 8: Survey T2 MAs translated**

| **A. Interventions of the CHANGE-3-study** | | | | | | |  |
| --- | --- | --- | --- | --- | --- | --- | --- |
| **I got to know the following CHANGE-3 interventions and they were utilized in practice:** | | | | | | | |
| 1) Mail  2) Mail for professional audience  3) Individualized data-based feedback report regarding antibiotic prescription  4) Outreach visit  5) Website (weniger-antibiotika.de)  6a) Patient information flyer regarding different acute, uncomplicated infections, German  6b) Patient information flyer regarding different acute, uncomplicated infections, foreign language | 7) E-learning training program regarding patient centred communication  8) Tablet PC containing patient information  9) Hatschi (colouring book)  10) Hatschi plush toy  11) COLD-magazine  12) Comic addressing school children | | | | | | |
| **The following CHANGE-3 interventions provided new impulses (input, knowledge, strategies) concerning the treatment of patients with acute, uncomplicated infections:** | | **Disagree**  **Strongly** | **Disagree** | **Neutral** | **Agree** | **Agree**  **Strongly** | |
| 1) Mail | |  |  |  |  |  | |
| 2) Mail for professional audience  (online and print) | |  |  |  |  |  | |
| 3) Individualized data-based feedback report regarding antibiotic prescription | |  |  |  |  |  | |
| 4) Outreach visit | |  |  |  |  |  | |
| 5) CHANGE-3-Website (weniger-antibiotika.de) | |  |  |  |  |  | |
| 6a) Patient information flyer regarding different acute, uncomplicated infections, German | |  |  |  |  |  | |
| 6b) Patient information flyer regarding different acute, uncomplicated infections, foreign language | |  |  |  |  |  | |
| 7) E-learning training program regarding patient centred communication | |  |  |  |  |  | |
| 8) Tablet PC containing patient information | |  |  |  |  |  | |
| 9) Hatschi (colouring book) | |  |  |  |  |  | |
| 10) Hatschi plush toy | |  |  |  |  |  | |
| 11) COLD-magazine | |  |  |  |  |  | |
| 12) Comic addressing school children | |  |  |  |  |  | |

| A-1: The website „www.weniger-antibiotika.de“ | Disagree Strongly | Disagree | Neutral | Agree | Agree Strongly |
| --- | --- | --- | --- | --- | --- |
| ... was visited by me |  |  |  |  |  |
| - If you „disagree strongly“ please skip to **A-2** |  |  |  |  |  |
| ... contains current information which is new to me |  |  |  |  |  |
| ... informs me in an understandable manner |  |  |  |  |  |
| … I consider helpful in patient communication |  |  |  |  |  |
| ... motivates me in supporting the GP more intensely in treating patients with acute respiratory tract infections |  |  |  |  |  |
| … strengthens the trust in my own skills regarding the participation in patient care. |  |  |  |  |  |
| … gives me confidence regarding the communication with patients |  |  |  |  |  |
| … influences to what degree I participate in the care of patients with acute respiratory tract infections. |  |  |  |  |  |

| A-2: The offered patient information material (poster, flyer, colouring book) | Disagree strongly | Disagree | Neutral | Agree | Agree Strongly |
| --- | --- | --- | --- | --- | --- |
| … is used in our practice |  |  |  |  |  |
| - *If you „disagree strongly“ please skip to* ***B-1*** |  |  |  |  |  |
| … informs in an understandable manner |  |  |  |  |  |
| ... reach the patients |  |  |  |  |  |
| … contain current information with regard to the utilization of antibiotics |  |  |  |  |  |
| … can facilitate the health literacy of patients |  |  |  |  |  |
| … can support the patient communication |  |  |  |  |  |
| … motivates me in supporting the GP more intensely in treating patients with acute respiratory tract infections |  |  |  |  |  |
| … strengthens the trust in my own skills regarding the participation in patient care |  |  |  |  |  |
| … gives me confidence regarding the communication with patients |  |  |  |  |  |
| … influences to what degree I participate in the care of patients with acute respiratory tract infections |  |  |  |  |  |

| B. Patient expectations | | | | | |  |
| --- | --- | --- | --- | --- | --- | --- |
| B-1: If antibiotics are prescribed, patients with acute respiratory tract infections think that | Disagree Strongly | Disagree | Neutral | Agree | Agree Strongly | |
| … the duration of consultation is reduced |  |  |  |  |  | |
| ... only one visit in practice is necessary in respect to the current infection |  |  |  |  |  | |
| ... their symptoms are alleviated faster |  |  |  |  |  | |
| … they can go back to work faster |  |  |  |  |  | |
| … they need antibiotics in the next infection |  |  |  |  |  | |
| … they can be satisfied with the medical treatment |  |  |  |  |  | |
| … they should better go to a physician who does not prescribe antibiotics |  |  |  |  |  | |
| ... they should better go to an alternative practitioner who does not prescribe antibiotics |  |  |  |  |  | |
